# Finding multifaceted communities in multiplex networks

**DOI:** 10.1038/s41598-024-65049-6

**Published:** 2024-06-24

**Authors:** László Gadár, János Abonyi

**Affiliations:** HUN-REN-PE Complex Systems Monitoring Research Group, University of Pannonia, Veszprém, Hungary

**Keywords:** Statistics, Applied mathematics, Information technology

## Abstract

Identifying communities in multilayer networks is crucial for understanding the structural dynamics of complex systems. Traditional community detection algorithms often overlook the presence of overlapping edges within communities, despite the potential significance of such relationships. In this work, we introduce a novel modularity measure designed to uncover communities where nodes share specific multiple facets of connectivity. Our approach leverages a null network, an empirical layer of the multiplex network, not a random network, that can be one of the network layers or a complement graph of that, depending on the objective. By analyzing real-world social networks, we validate the effectiveness of our method in identifying meaningful communities with overlapping edges. The proposed approach offers valuable insights into the structural dynamics of multiplex systems, shedding light on nodes that share similar multifaceted connections.

## Introduction

There are several algorithms to divide networks into groups to identify structural elements that are more likely to be connected to each other than the rest of the network^1^. However, none of them considers the optimization of overlapping edges within a cluster. The best representation of cooperating systems with interacting actors are networks, which are systematically multilayered due to the variety of connections^2^ in social networks^3^, author networks^4^ and organizational networks^5^. Among many network properties, the exploration of the mesostructure is a fruitful area of research to describe the characteristics of interactions. The emergence of relationships is not random, which is why it gives us the opportunity to investigate the deviation from chance when communities are obtained^6^. Modules are formed for various reasons because the likelihood of link formation between set of actors is influenced by some cost function. One of the most obvious cost factors is the physical distance^7,8^ in geographically distributed networks^9^, as well as when nodes tend to connect to similar^10^ or adventive peers^11^.

There are several algorithms for separating modules^12^, and in this article we present a modularity measure that separates communities by the overlapping edges in multilayer networks. The heart of the procedure is the calculation of the relative strength of the link in the modularity matrix^13^. Most often, the modularity matrix represents the number of actual connections relative to the expected values defined by a random consideration, which is used in Louvain^14^ or Leiden^15^ algorithms to detect modules. It has been further refined using multiresolution methods (e.g., RB^16^, AFG^17^) to detect smaller modules to improve the resolution limit^18^, which may have a community stability limitation^19^.

The methodological toolbox for exploring modules relative to random connections has been significantly extended with the modification of the negative term (the null network) to calculate the modularity matrix. Module discovery is carried out considering a goal-oriented baseline^20^, a benchmark, a null model^21^, or a penalty factor^22,23^, which has almost the same objective even in multilayer networks^24^. The negative term in the calculation of the modularity matrix is actually a null network, and the same null network can correspond to different null models^25^. In multilayer networks, the following strategies are used to obtain the community structure^26^: (1) collapse of layers into a single layer network, then the algorithm is applied^27^, (2) a single layer module search is performed on each layer, and then the communities obtained per layer are combined to form a consensus^28^, (3) a direct method simultaneously detecting both cross-layer and single-layer communities^22^. The performance of various community detection algorithms on multilayer networks can be evaluated by their capability to identify ground truth communities, the similarity of community structures obtained by different algorithms, and their scalability^29^. It should be highlighted here, our objective is not to devise a new algorithm or identify ground truth communities. Instead, we aim to uncover meaningful communities that can be explored through overlapping edges. Consequently, the similarity to ground truth and the scalability are not considered as metrics.

Our goal is to explore communities in which individuals share similar multifaceted relationships. This means that they form a network structure that is characterized by a greater likelihood of being connected with multiple edges within the community than with other actors in the network. There are solutions using generative models for the mesostructure in multilayer networks^30^. However, analyzing groups of nodes in a complex system characterized by multifaceted relationships can be challenging for model-based community detection algorithms due to the intricate interplay between network layers. The emergence of communities is often based on similarity in attributes or continuous variables shared between vertices^31^, and the number of similarity criteria increases with the number of layers in the network. We use one or more layers of the network as a null network or reference for module discovery, rather than a model that approximates random relationships as expected connections. Thus, we exploit the wide range of information in multilayer networks.

In our approach, to achieve the aim, the modularity matrix is calculated with a positive (first) factor that defines the network under study, while the negative (second) term provides a reference against which we identify excess connections between nodes *i* and *j*. This simple framework facilitates the discovery of meaningful communities characterized by overlapping edges. With our novel modularity measure for multiplex networks, we aim to contribute to a deeper understanding of community structures. Although some argue that completely novel insights into community structures are rare^32^, our approach offers a fresh perspective by focusing on overlapping edge groups. However, the absence of gold standard communities^33^ poses challenges, as the relative nature of the node groups complicates the optimization process for maximal modularity. Our exploratory module detection method operates within the constraints imposed by the null network. The modularity matrix can be used with any modularity optimization algorithm, and in this work, we employ the Louvain algorithm^14^. It is not our aim to compare and test other numerical optimization solutions to maximize modularity, but only to investigate the influence of the null network on the module structure. In this paper, we demonstrate how substituting a null network with a network layer leads to the emergence of new communities, with a focus on the degree of edge overlap within these communities.

**Figure 1:**
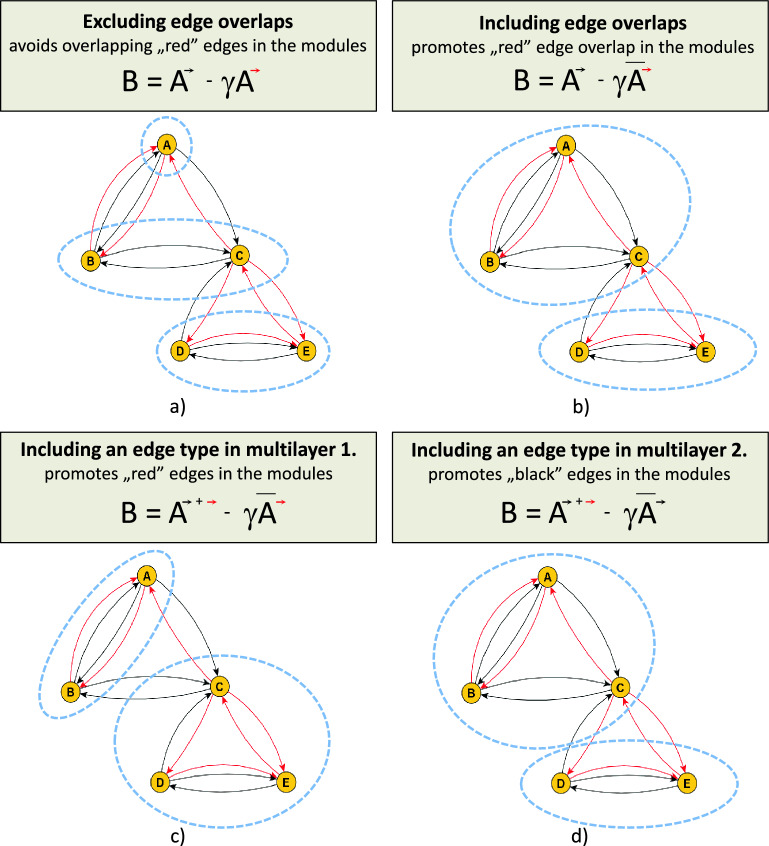
The simplified introduction illustrates the application of one network layer as a null network in obtaining the community structure of an example multiplex network, where black edges represent connections in one network layer and red edges represent connections in another network layer. The variability of the null network in the modularity matrix leads to different meaningful module structures provides by edge overlap. the code for the community detection illustrated in the figure is available on the github page for this article^34^. (*B*: modularity matrix, *A*: adjacency matrix, ${\bar{A}}$ means the complement graph of adjacency matrix (0 if connected, 1 if disconnected in the original graph).

The contribution of the community detection technique presented is its ability to prefer or avoid edge overlaps within communities empirically. It categorizes similarly connected elements into a group based on the multifaceted relationship between them. By including or excluding edge overlaps within modules, we can reveal communities with special meanings. For example, we may identify working communities with a friendship surplus between members of an organization. In the graphical abstract shown in Fig. 1, we illustrate the operation of modularity matrix which apply a network layer as null network for a simplified two-layer network. In the first case (Fig. 1a), the modification of the modularity matrix (B) aims to minimize the number of red edges in communities adjacent to the black edges. In the second case (Fig. 1b), the application of the complement graph attempts to include overlapping red edges within communities. In the bottom row, the third and fourth cases (Fig. 1c,d) illustrate the ability of the technique to prefer the inclusion of specific layers within communities in the context of a multilayer and weighted merged network. The method effectively groups similar elements based on their shared multifaceted relationships is shown with real world multilayer networks in the article.

In the “Methods” section, we describe and formalize in detail how we investigate the relational surplus with respect to a layer of a multiplex network as a null network. In the “Results” section, we give empirical examples of how the methodology works on a real world network. The methodology is demonstrated in a multiplex organizational social network with different aspects of link types consisting of 67 employees and leaders. In the first subsection, we identify coworking communities with low probability of having trustfully friendship ties and are therefore potential threats to good collaboration. In the second subsection, we explore collaborative professional communities in which the professional knowledge of members is highly evaluated indicating a healthy professional cooperation between coworkers. In the third subsection, we evaluate the similarity of community structure obtained by the preference of a network layer in modules compared to the traditional community detection method, thus inferring similar community-shaping forces. In the last section, we conclude our experiences with the empirical null network. The method was tested on an additional open organizational network (AUCS network^35^) and on a large-scale network (Erasmus student network^36^) presented in the Supplementary materials.

## Method to find multifaceted communities

After describing the network used, we present three methods that explore multifaceted communities in different ways by choosing an appropriate null network. In the first procedure, we employ the corresponding null network to investigate communities in which members are connected through links present in the observed network layer while avoiding connections from other layers, as illustrated in Fig. 1a. In the second procedure, we achieve the opposite by using the complement graph of the null network, forming communities with a high probability of containing null network connections in addition to the connections in the observed network layer, as depicted in Fig. 1b. In the third application, we use the weighted network obtained by merging the multilayer network to uncover communities where members exhibit a higher likelihood of interconnectivity through multifaceted edges compared to the rest of the network, as depicted in Fig. 1c,d.

### The network

In the organizational network which is used to demonstrate the method, nodes are connected with labeled edges, reflecting how the members connected to work together, consider being a friend, get professional advice and evaluate each other’s high professional knowledge. Labeled and directed links define multidimensional edges and form a multidimensional network ${\mathcal {G}} = (V, E, D)$, where *V* represents the set of nodes, *D* the set of edge labels defines the dimensions of the edges, and *E* denotes the set of edges, $E = \{(u, v, d); u, v \in V, d \in D\}$. In the directed graph, the edges (*u*, *v*, *d*) and (*v*, *u*, *d*) are distinct.

As each edge label can be assigned to an independent network, the model can be interpreted as a multilayer network. A multilayer network is a pair ${\mathcal {M}} = ({\mathcal {G}},{\mathcal {E}})$, where ${\mathcal {G}} = \{G_\alpha ; \alpha \in \{1,..., M\}\}$ is a family of graphs $G_\alpha = (X_\alpha , E_\alpha )$ (called layers of ${\mathcal {M}}$) and ${\mathcal {E}} = \{E_{\alpha \beta } \subseteq X_\alpha \times X_\beta ; \alpha , \beta \in \{1,..., M, \alpha \ne \beta \}$ is the edge set between nodes of different layers $G_\alpha$ and $G_\beta$ with $\alpha \ne \beta$^37^. $E_\alpha$ are called intralayers and $E_{\alpha \beta } (\alpha \ne \beta )$ are referred to as interlayer-connections. In this study there are no interlayer connections. In our case, the multidimensional network $G = (V, E, D)$ is associated with a multiplex network with layers $G_1,..., G_{|D|}$ where $\alpha \in D,\, G_\alpha = (X_\alpha , E_\alpha ),\, X_\alpha = V,\, E_\alpha = \{(u, v) \in V \times V; (u, v, d) \in E \; \text {and} \; d = \alpha$}. Nodes are permanent actors; thus, the network is multiplex, which is a special case of multilayer networks.

Consider an ${\mathcal {M}}$ multiplex network with N nodes and let the edge weights between pairs of nodes be $\{A_{ij}|i,j \in \{1,...,N\}\}$, that is, $\varvec{A} = (A_{ij}) \in {\mathbb {R}}^{N \times N}$ is the adjacency matrix of ${\mathcal {G}}_\alpha$. The adjacency matrix is asymmetric in our case because the network is directed, so $A_{ij} \ne A_{ji}$.

Different layers of the network are used to demonstrate the methodology presented below. *In the first example* one of the layers of the network represents the cooperation relationship between members of an organization. A directed edge from node *i* to node *j* represents the situation where node *i* has to work together with node *j* frequently. The second layer of the network is friendship, where the set of nodes is the same, and there is an edge from node *i* to node *j* if node *i* considers node *j* a friend.

*In the second example* the two layers of the network seek professional advice and graded professional knowledge, where both layers of the network are a directed network. The advice network represents relationships in which node *i* starts a directed edge to node *j* when seeking professional advice from colleague *j*. In the network layer rating node *i* starts an edge to node *j* if *i* considers *j* to have a high professional knowledge in their own field of profession.

*In the third example* three layers of the network are used that represent a complex cooperation system of members of an organisation. The coworkers, the friendship, and the professional advice networks were merged into a weighted and directed network, in which the weights are proportional to the number of dimensions. The maximum number of weights (three) means that node *i* connects to node *j* in all types of connections. The weight of 2 means that one of the relationship types is missing in the link from node *i* to node *j*, and we know exactly which one. A weight of 1 means that node *i* is connected to node *j* by one of the three types of links. The network layers form a network on their own with the same set of nodes.

### Community structure in general

For studying the community structure of a network, we compare the observed network with an expected network in general. Null models and null networks are not exactly the same. One defines a null model as a probability distribution on the set of adjacency matrices and a null network as the expected adjacency matrix under a specified null model^25^. Null models serve as prior models by accounting for anticipated features within the system under investigation. They allow the consideration of known or suspected connectivity patterns that could potentially mask undiscovered connectivity patterns. It is common to consider the degree (or strength) of nodes in the null network as they are taken in the observed network, in social networks^6^. This consideration reflects the fact that connectivity is determined by the strength of the vertices, and the modularity function seeks connectivity surpluses regarding the null network, in the case of the vertex degree/strength. In spatial networks, nodes are geographically distributed, and the probability of links between them decreases with geographical distance^7^, except if the connection cost is financed by a third party^38^. The authors used null models considering spatial cost to uncover spatially independent communities^9,39^. Their null model allows the exclusion of the known spatial location attribute in order to explore the unknown vertex property (e.g. homophilic relations).

Classical modularity optimization-based community detection methods utilize *f*(*C*) metrics that are based on the difference between the internal number of edges and their expected number^6,39^.

1 $$\begin{aligned} f(C) = \text {(fraction of edges within communities)} - \text {(expected fraction of such edges)}. \end{aligned}$$

Modularity measures the surplus of connections in the observed network compared to the null network. Mathematically, the modularity of a partition *C* in a directed network is calculated as

2 $$\begin{aligned} Q (C|\varvec{A},\varvec{P}) = Q_C = \frac{1}{L} \sum \limits _{i,j} \left( A_{ij}-P_{ij}\right) \delta \left( c_i,c_j\right) \end{aligned}$$

where $\delta (c_i, c_j)$ is the Kronecker delta, which is 1 if nodes *i* and *j* belong to the same module and 0 otherwise. Given the adjacency matrix $\varvec{A} = (A_{ij}) \in {\mathbb {R}}^{N \times N}$ of the observed network and the adjacency matrix $\varvec{P} = (P_{ij}) \in {\mathbb {R}}^{N \times N}$ of the reference null network, then $Q_C$ is the modularity for partitioning $C\in {\mathcal {C}}$, when the *i* and *j* nodes belong to the same *C* community. The $\varvec{B} = \varvec{A} - \varvec{P}$ is the modularity matrix^40^.

Suppose that we have a community structure ${\mathcal {C}}$ of the network containing K disjoint sets of nodes {$C_1$, ..., $C_K$}. We can define a mapping function, denoted as c(.), that assigns nodes of the set $\{1,\ldots , N\}$ to integers in the set $\{1,\ldots , K\}$ indicating the community. This mapping satisfies the condition $c(i) = c(j) = k$ if and only if the nodes *i* and *j* belong to the same $C_k$ community^25^. The modularity value of *C* can be positive, negative, or zero. Should it be equal to zero, when the community has as many links as the null model predicts, positive if the fraction of edges within communities is higher than the null model predicts, and negative otherwise. The positive modularity of the community indicates a more inner connection relative to the attribute of the null model. Despite the fact that there is a surplus of relationship information encoded by the null model, e.g. geographical distance. The modularity of the observed directed network^1^ for partitioning ${\mathcal {C}}$ is

3 $$\begin{aligned} Q = \frac{1}{L} \sum \limits _{C \in {\mathcal {C}}} \sum \limits _{ij \in C} \left( A_{i,j}-P_{i,j}\right) \delta \left( c_i,c_j\right) \end{aligned}$$

where $i,j \in C$ is the sum of pairs of nodes *i* and *j* belonging to the same community $C \in {\mathcal {C}}$; *L* = $\sum A_{ij}$ is the total number of weights in the network. In general, the observed and null network satisfy $\sum A_{ij}$ = $\sum P_{ij}$ = *L* for all *i* and *j*. When an algorithm classifies vertices into modules, it defines communities by maximizing the value of modularity (Q) in Eq. (3) without predefining how many modules the network contains. The number of *K* sets of communities depends on the optimization problem. If $A_{ij} < P_{ij}$ for all *i* and *j* connections, then the optimal solution is N singleton communities, and if $A_{ij} > P_{ij}$ for all *i* and *j* links, then the optimal solution is a single N node community^25^. Underestimated link probabilities are the sources of positive modularity, and when the null model more closely approximates the observed network, the modularity is expected to be lower^8^.

The probability of connection between any nodes *i* and *j* is described in $\varvec{P}$, which is a chance of link under the estimation of the null model. It is often a random network with certain constraints that correspond to known information^39^. Different null models can be created depending on the specific network being studied and the research objectives^32^. The most widely applied null model is the random configuration model, exactly the Chung-Lu model^41^, which calculates the edge probabilities assuming a random graph conditioned to preserve the degree sequence of the original network, as

4 $$\begin{aligned} P_{ij}^{NG} = \frac{k_i k_j}{2m} \end{aligned}$$

where $k_i$ and $k_j$ are the degree if node *i* and *j*.

Consequently, NG communities are obtained by maximizing the modularity described by the following equation.

5 $$\begin{aligned} Q^{NG} = \frac{1}{L} \sum \limits _{C \in {\mathcal {C}}} \sum \limits _{ij \in C} \left( A_{i,j}-\frac{k_i k_j}{2m}\right) \delta \left( c_i,c_j\right) \end{aligned}$$

The modularity function method is motivated by the idea that a network partition should be considered meaningful when there are more edges between nodes of the same group than would be expected in a random null model without communities^42^. The algorithm is blind to communities consisting of less than $\sqrt{L/2}-1$ internal links^18^ and the problem was handled with improving the resolution limit, for example, with the RB^16^ and AFG^17^ methods.

A good example of the variability of the null model is the uncovering of space-independent communities where the model considered the importance of the node and the empirically determined distance-dependent deterrence function^39^:

6 $$\begin{aligned} P_{ij}^{Spa} = N_i N_j f(d_{ij}) \end{aligned}$$

where $N_i$ is a notion of importance of node *i* (such as the degree or GDP of a city if the nodes are cities) and $d_{ij}$ is the empirically determined distance-dependent deterrence function. Subtracting the property known by the null model from the real network allowed clustering based on another property (language-based similarity).

This example illustrates how the community detection algorithm identifies similar elements and groups them into communities using the modularity matrix based on existing information coded in a null network as a viewpoint. The selection of this perspective impacts the resultant communities, which are then subjected to an in-depth analysis to gain insight into the network. The variability of the null network is utilized effectively in the network layer approach introduced in the next subsection.

### Proposed null networks for finding multi- or single faceted communities: a multiplex network layer

Two main factors are encouraged to propose null networks. (1) The modularity of a network is measured by the deviations from well-mixed *random* expectations to understand their properties as introduced by Ref.^43^. But the null model is varied to be goal-oriented to control a feature of the observed network to conclude a phenomenon. The variability of null models improves researchers in attaining inferential criterion, however, it has limitations^42^. One lesson is that by selecting an appropriate viewpoint provided by the null model or benchmark graphs, structurally similar nodes belong to the modules differently^44^. (2) Multifaceted relationships between actors increase the complexity of networks. The uncovering of communities in multilayer networks is challenging, and there are several solutions. One of the problems in the construction of null models is the large number of possible empirical features in multilayer networks^30^, and the other is the interrelated connection between layers^45^, but it is not relevant in our example. Another lesson is that the range of possibilities to obtain the community structure of multilayer networks is growing as information grows.

Taking advantage of the opportunity to change the benchmark, we propose using the following null networks to make better use of the information available by multiplex network layers. In contrast to synthetic and/or random null networks, we suggest and test in this paper that one of the empirical layers of the network be included as a viewpoint to the modular structure of the network. In this way, a network layer has a new role in multilayer networks. The null model does not assume to be measured by a benchmark under random consideration. The null network, which is also a network layer, is empirical information that contributes to the meaningful mesostructure of a multilayer network. A different community structure is formed depending on the variability of null network, and we can test the affect and similarity of each layer information on the community structure.

The advantages of using the network layer as a null network are as follows.

- The method allows for the exploration of smaller groups of nodes with similar connectivity patterns, providing deeper insights into the most important patterns regarding the functions and mechanisms driving link formation.
- The null network is an empirical network, correlated with the observed network^5,46, 47^, a property that is exploited in this article. The method is obviously influenced by the degree of overlap of the edges between layers. We take advantage of the fact that edge overlap is not uniformly and randomly distributed throughout the network, and communities will reflect this.
- The aim of the presented method is not to find the ground-truth community structure. It is about finding meaningful communities with a specific purpose.
- By changing the null network, which is a network layer, the expert can control the meaning of modules promoting or avoiding layer overlaps in communities of multiplex networks. This allows domain experts to intentionally study the community structure of the network.
- The method is independent of the modularity maximization algorithm, since only the null network is modified. It can be used with any numerical optimizer.

Considering edge overlap, there are three possible cases for edges between nodes *i* and *j* (shown in Table 1): (1) there is an edge in the observed network layer and there is another edge in the null layer (2) there is an edge in the observed network layer and there is no edge in the null layer, (3) there is no edge in the observed network layer and there is an edge in the null layer. The fourth possible case that there is no edge between vertices in either layer is irrelevant. Consequently, when a deviation from the null network layer is calculated, the modularity matrix will have values of (1) moderately decreasing the edge weight of observed network, (2) remaining the same as in the observed network, and (3) drastically decreasing and making the edge weight below zero, respectively. The affect of the null network layer can be adjusted with the ($\gamma$) parameter when calculating the modularity matrix.

**Table 1:** Cases of connectivity surplus in the observed network layer compared to the null network layer. In practice, the values of $P_{ij}$ are 0 and 1 if the density of the observed network and the null network are the same, see Eq. (13).

$A_{ij}$	$P_{ij}$	$B_{ij}$	Explanation
1	1	0	There is no connectivity surplus compared to the null network layer
1	0	1	High connectivity surplus compared to the null network layer
0	1	$-1$	High connectivity deficit compared to the null network layer
0	0	0	Irrelevant.

Three types of null networks are presented below that can be used for different tasks.The formalized modularity matrices $\varvec{B} = \varvec{A} - \varvec{P}$ are described below.

*The first case*, when the modularity matrix guides the algorithm to find communities that avoid edge overlap in clusters, is the following.

7 $$\begin{aligned} \varvec{B}^{exclusion} = {\left\{ \begin{array}{ll} \varvec{A} = A_{ij}^{l_k} \\ \varvec{P} = \gamma \, A_{ij}^{l_{m \ne k}} \end{array}\right. } \end{aligned}$$

where the observed network layer is denoted by $l_k$ and the null network is denoted with $l_m$ are in the same ${\mathcal {G}}$ multiplex network, where $m \ne k$, and $G_{l_k}, G_{l_m} \in {\mathcal {G}}$. Equation (7) attempts to exclude the edges of the $l_m$ layer along the edges of the $l_k$ layer when communities are obtained. Taking into account Eq. (3), the modularity of the network is denoted by $\varvec{Q^{exclusion}}$ in this work, reflecting that a pair of network layers is used.

In our example in the “Results” section, we look at communities of colleagues in a workplace. It is reasonable to assume that work relationships combined with friendship tend to be stronger and more trustworthy^48^. We are looking for communities where the friendship layer overlaps with the coworker network layer is less likely, indicating that members collaborate but are unlikely to be friends. This information supports the leader, who can expect that members in such communities probably distrust each other, and the situation may lead to rivalry or conflict.

*In the second case* the modularity matrix helps in finding communities where relationships are multidimensional and excludes those where connections are simpler as follows:

8 $$\begin{aligned} \varvec{B}^{inclusion} = {\left\{ \begin{array}{ll} \varvec{A} = A_{ij}^{l_{k}} \\ \varvec{P} = \gamma \, {\bar{A}}_{ij}^{l_{m \ne k}} \end{array}\right. } \end{aligned}$$

where ${\bar{A}}_{ij}^{l_{m \ne k}}$ is the adjacency matrix of the complement graph of the $G_{l_m}$ network, that is,

9 $$\begin{aligned} {\bar{A}}_{ij}^{l_{m}} = {\left\{ \begin{array}{ll} 0 \text { if the original } A_{ij}^{l_{m}} = 1 \text { meaning nodes i and j are connected},\\ 1 \text { otherwise.} \end{array}\right. } \end{aligned}$$

In graph theory, the complement graph of a given graph is a new graph that has the same set of vertices, but edges connecting pairs of vertices that are not connected in the original graph. The complement graph is obtained by removing the existing edges and adding the missing edges^49^. The complement graph was used to the balanced Max-Cut NP complete problem to obtain modules in a multiplex by analyzing the edges between partition classes^50^. Another application was the creation of a measure called Max–Min modularity to minimize unrelated pairs of nodes in the same community^51^. Here, we introduce a novel application of complement graphs to obtain communities with layer overlap.

The null network $\varvec{P}$ is a matrix in which the values 0 and 1 are the complement graph of the original rating network, that is, if node *i* evaluates *j* with high professional knowledge, then the edge between *i* and *j* ($P_{ij}$) has a weight 0 and otherwise 1. Using Eq. (8), the adjacency matrix of the complement graph as a null network reveals communities in which the relations within the module tend to be multidimensional. In this case, the null network ”penalizes” the missing edges of the original (not complement) null network. Equation (8) promotes the emergence of communities in which the edge dimension coded by the null network layer is more likely to occur together at the edges of the observed network layer and attempts to include the edges of the $l_m$ layer along the edges of the $l_k$ layer when communities are obtained. Considering the Eq. (3), the modularity of network is denoted with $\varvec{Q^{inclusion}}$ here, reflecting that a complement graph of the network layer is used as null network.

In this article, we show how the formula works on a two-layer network in the Results section. The network under study is a directed network of professional advice ($G_{l_k}$) from coworkers. The null network is the complement graph of the high professional knowledge rating network ($G_{l_m}$). Our aim is to uncover professionally cooperative communities in which members probably value the professional knowledge of their collaborating partners, which is a source of healthy professional cooperation.

*In the third case*, we want to identify the layer with the greatest effect on the traditional NG community structure in a merged multilayer weighted and directed network. The network under consideration is a multilayer merged network with edges that are directed and weighted. The null network is formed by taking one or more layers of the observed weighted network and forming its weighted complement graph as follows.

10 $$\begin{aligned} \varvec{B}^{multi} = {\left\{ \begin{array}{ll} \varvec{A} = A_{ij}^{l_{1+2+ \cdots +m}} \\ \varvec{P} = \gamma \, {\bar{A}}_{ij}^{l_{k+ \cdots +l}}, \text { where } k \le m \text { and }l \le m \end{array}\right. } \end{aligned}$$

where the null network layer(s) is (are) part of the combined multiplex network, ${\mathcal {G}} = \{G_{l_i}; i \in 1,..., m\}\}$ and we study the contribution of $G_{l_k}$ (or $G_{l_k} + \cdots + G_{l_l}$ combined multilayer network) to the traditional NG community structure of ${\mathcal {G}}$. The complement graph concept needs to be adjusted in the weighted case. The matrix representation of the original network layer is conversed similarly than in Eq. (8), because in this way we can promote overlapped relationships and penalize non-overlapped relationships in the modularity matrix. Since the adjacency matrix of the null network is subtracted from the adjacency matrix of observed network when calculating the modularity matrix, the promotion means that the edge in the original null network with the maximum number of dimensions should be 0, while the penalty is performed by converting the missing connections to the maximum of the edge weights in the complement graph. For links with a nonmaximum number of dimensions, the edge weight of the complement graph will be the residual with respect to the maximum. The edge weights of the complement graph in weighted case is the following.

11 $$\begin{aligned} {\bar{A}}_{ij}^{l_{k+ \cdots +l}} = max_{ij}(A_{ij}^{l_{k+ \cdots +l}}) - A_{ij}^{l_{k+ \cdots +l}} \end{aligned}$$

For a pair *i* and *j*, $A_{ij}^{l_{k}}$ can be 0 or 1, but $max_{ij}(A_{ij}^{l_{k+ \cdots +l}})$ is the maximum value of the matrix that describes the merged multiplex network. The modularity of the network is denoted by $\varvec{Q^{multi}}$ in this article, reflecting that a merged multilayer network is used.

Since the network does not have a ground truth community structure and our objective is not to identify it, we defined NG communities of merged weighted multilayer network as a reference by maximizing $Q^{NG}$ and observed the extent of change in the mesostructure when maximizing $Q^{multi}$. The similarity in the community structure is measured with the traditional Normalized Mutual Information (NMI) proposed by Ref.^52^ as follows considering the confusion matrix.

12 $$\begin{aligned} NMI(X,Y) = \frac{-2 \sum _{i=1}^{c_X} \sum _{j=1}^{c_Y} N_{ij} log(\frac{N_{ij}N}{N_{i.} N_{.j}})}{\sum _{i=1}^{c_X} N_{i.} log(\frac{N_{i.}}{N}) + \sum _{j=1}^{c_Y} N_{.j} log(\frac{N_{.j}}{N})} \end{aligned}$$

where X and Y are the class labels of the nodes, $c_X$ and $c_Y$ the number of communities found, the sum of row *i* of the confusion matrix $N_{ij}$ is denoted $N_{i.}$ and the sum of the column *j* is denoted $N_{.j}$. The function assesses the distance between two community structures. If the community structure denoted by *X* and *Y* is the same, then $NMI(X,Y) = 1$, and if completely different, then $NMI(X,Y) = 0$.

We assume that the information of a complement network layer as null network that less changes the community structure of NG means that the two viewpoints are close to each other.

The overlapped network layers are not equal in terms of density and number of vertices involved. Also, if edges are present in one layer, they may not be present in the other layer. In all cases, $\gamma$ is the adjustment parameter that controls the strength of the null network consideration. Similarly to other community structure algorithms, as is expected from the null network, to fulfill the following equality:

13 $$\begin{aligned} \sum \limits _{i,j} A_{ij} = \sum \limits _{i,j} P_{ij} = L \end{aligned}$$

where L is the number of edges in the directed network. Taking Eq. (13) into account ensures that the observed and null networks are regarded with the same weight and the difference in average weight does not affect the detection of the community.

To determine the community structure, a greedy Louvain algorithm^14^ was used that is implemented in R^53^ as part of the NetworkToolbox package^54^ with little modification. Modifications to the cited function included the optional change of the modularity matrix as input and expanded the output parameters to calculate the modularity of the modules. All codes and databases used in this article are freely available^34^ to ensure reproducibility and transparency.

The proposed null network modification method with the Louvain algorithm was evaluated on three networks, two containing fewer than 100 nodes, and a network with close to 2000 vertices. The scaleability of our method is the same as that of the Lovain algorithm. Our experience revealed that the algorithm’s performance deteriorated as the size of the larger network increased. Specifically, the runtime on the small network was negligible when executed on a local PC (Intel(R) Core(TM) i7-2600 CPU @ 3.40GHz, 3401 Mhz processor, with RStudio in Windows 10), while on the larger network, it took approximately 10 s.

### Ethical statement

Participation in the investigation required the completion of a questionnaire. Respondents voluntarily participated in the research and they were informed consent prior to their participation. The authors declare that there are no ethical issues with the results presented. The research was carried out following the procedures outlined in the Declaration of Helsinki. All research participants worked according to the protocols declared in the Code of Ethics of the University of Pannonia, Veszprém, Hungary. Ethics approval by the institutional committee is not required specifically for this research.

## Results and discussion

The results are demonstrated using a real-world multilayer network of an organization where 67 coworkers are connected in various aspects. The network layers represent the relational dimensions between the members and leaders of an organization as follows.

- $l_1$: cooperation with colleagues,
- $$l_2$$: friendship,
- $$l_3$$: professional advice,
- $l_4$: evaluation of high professional knowledge.

The three applications outlined below address practical challenges. In the first application, we identify communities where members collaborate ($l_1$) without considering the peer a friend and sharing the same beliefs or interests ($l_2$). Collaborative communities without trust capital can be a threat to harmonious relations. In the second application, we focus on professional communities where members seek advice from colleagues ($l_3$) while valuing their professional knowledge ($l_4$). In the third application, we analyze the merged collaborative network created by combining the layers of collaboration, friendship, and professional advice ($l_1$, $l_2$, $l_3$) to determine which edge overlap promotion within the modules is closest to the NG community structure of the merged network.

### Uncover communities with exclusion of overlapping edges

**Figure 2:**
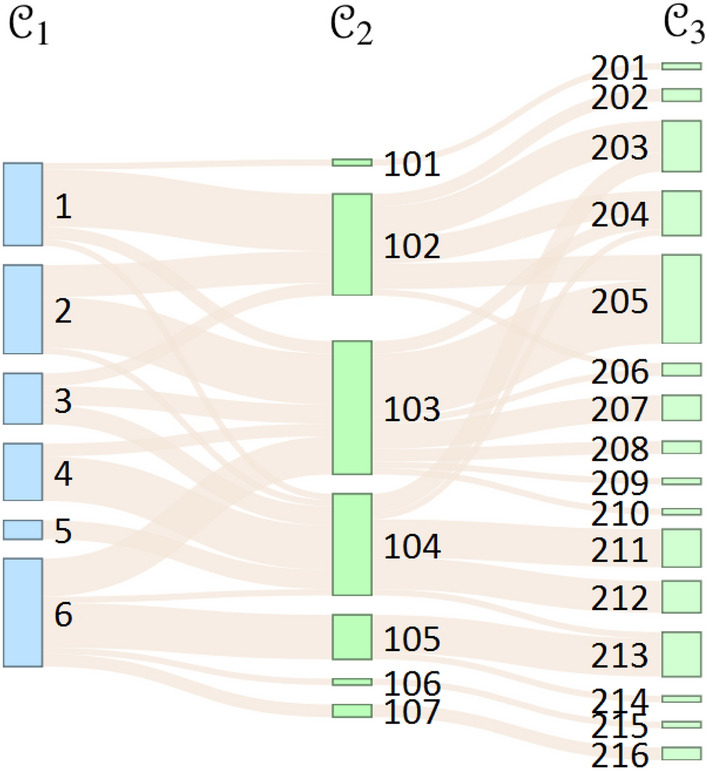
Transformation of communities explored by different null models. The size of the lanes is proportional to the number of people. The observed network layer is $l_1$: cooperation network. The null network in community structure ${\mathcal {C}}_1$ (blue) obtained with the configuration model (Eq. 5). In the community structure ${\mathcal {C}}_2$ (green) the null network is the friendship network layer ($l_2$) at $\gamma =1$ (Eq. 14), and in the community structure ${\mathcal {C}}_3$ (light green) the null network considered stronger at $\gamma =2$ (Eq. 14).

In the first approach, our goal is to obtain communities that have a lower probability of edge overlap. We prove that the null network referenced by Eq. (7) is capable of doing that. The null network weakens the modularity matrix at the elements for which it is true that the edges of the null network appear together with the edges of the observed network. That is, when maximizing modularity $Q^{exclusion}$, overlapping edges are given less weight in the modularity matrix.

14 $$\begin{aligned} \varvec{Q}^{exclusion} = \frac{1}{L} \sum \limits _{C \in {\mathcal {C}}} \sum \limits _{ij \in C} \left( A_{ij}^{l_1}-\gamma A_{ij}^{l_2}\right) \delta \left( c_i,c_j\right) \end{aligned}$$

where the observed network is the cooperation network of coworkers ($l_1$), and the null network is the friendship network ($l_2$) of the same set of nodes. In Fig. 2 and Table 2, we demonstrate that the approach discovers collaborative communities of coworkers in which the co-occurrence of friendships is excluded within modules as much as possible in the community structure ${\mathcal {C}}_2$ where $\gamma =1$ and ${\mathcal {C}}_3$ where $\gamma =2$.

**Table 2:** Characterisation of modules shown in Fig. 2. $Q_c$ is the local modularity of the module. The $P(l_2|l_1)$ means the probability of edges of null network that occur together with edges of observed network within modules.

${\mathcal {C}}_1$				${\mathcal {C}}_2$				${\mathcal {C}}_3$			
Module id	Number of members	$Q_c$	$P(l_2|l_1)$ within module	Module id	Number of members	$Q_c$	$P(l_{2}|l_{1})$ within module	Module id	Number of members	$Q_c$	$P(l_{2}|l_{1})$ within module
1	13	0.135	0.492	101	1			201	1		
				102	16	0.100	0.411	202	2	0.003	0.000
								203	8	0.023	0.273
								204	7	0.026	0.188
2	14	0.084	0.615	103	21	0.079	0.449	205	14	0.051	0.095
								206	2	0.003	0.000
								207	4	0.013	0.000
								208	2	0.003	0.000
								209	1		
								210	1		
3	8	0.059	0.714								
4	9	0.077	0.645	104	16	0.087	0.522	211	6	0.020	0.222
								212	5	0.022	0.273
5	3	0.019	0.500								
6	17	0.138	0.452	105	7	0.054	0.333	213	7	0.038	0.176
								214	1		
				106	1			215	1		
				107	2	0.003	0.000	216	2	0.003	0.000
		$Q^{{\mathcal {C}}_1}=0.512$				$Q^{{\mathcal {C}}_2}=0.323$				$Q^{{\mathcal {C}}_3}=0.195$	
$Q^{{\mathcal {C}}_1} = Q^{NG}_{l_1}= \frac{1}{L} \sum \limits _{C \in {\mathcal {C}}} \sum \limits _{ij \in C} \left( A_{i,j}^{l_1}-\frac{k^{l_1}_i k^{l_1}_j}{2m}\right) \delta \left( c_i,c_j\right)$				$Q^{{\mathcal {C}}_2} = Q^{exclusion, \gamma =1}_{l_1,l_2} = \frac{1}{L} \sum \limits _{C \in {\mathcal {C}}} \sum \limits _{ij \in C} \left( A_{ij}^{l_1} - 1 A_{ij}^{l_2}\right) \delta \left( c_i,c_j\right)$				$Q^{{\mathcal {C}}_3} = Q^{exclusion, \gamma =2}_{l_1,l_2} = \frac{1}{L} \sum \limits _{C \in {\mathcal {C}}} \sum \limits _{ij \in C} \left( A_{ij}^{l_1} - 2 A_{ij}^{l_2}\right) \delta \left( c_i,c_j\right)$			

As a crucial indicator, we have focused on the probability of edge overlap between the null network and the observed network within the modules. Given the absence of a ground truth module structure and our objective is not to identify it, we used the NG community structure as a reference for comparison (${\mathcal {C}}_1$), where $A_{ij}$ values come from the $l_1$ network layer and $P_{ij}$ values comes from the configuration model. We observed the connections between the grouped vertices in the $l_2$ network layer and calculated the edge overlaps within the module.

For instance, in Module 1, there is a probability of 0.492 that the edges of the null network overlap with the edges in the observed network. This indicates that within Module 1, roughly half of the observed network’s edges are adjacent to an edge of the null network. After partitioning Module 1 of ${\mathcal {C}}_1$ into Modules 101 and 102 of the community structure ${\mathcal {C}}_2$, calculated using Eq. (14), the probability of edge overlap reduces to 0.411 in Module 102. This transformation is achieved by excluding an actor from Module 1 to Module 101 which has relatively low in- and out-degrees, but every edge overlapped with edges from the null network. Additionally, two actors from Module 1 regrouped into Module 103 and one actor into Module 104. Regrouped actors have connections with nodes of other communities through edges of the observed network, but fewer connections in the null network layer. Consequently, the local modularity value of Module 102 remains high ($Q_c = 0.100$) while the edge overlap rate decreases. The further partition of Module 102 into Modules 202, 203, and 204 of ${\mathcal {C}}_3$ involves cuttings along the overlapping edges, retaining those edges between the modules that increase the overlap of the edges within the modules.

In conclusion, maximization of modularity $Q^{exclusion}$ is successful by reducing edge overlaps within modules. Maximization is achieved by reassigning network actors to other modules or by exclusion of them from the module. It is worth noting that using the null network to adjust the $\gamma$ parameter results in substantial interference with the community structure. This is attributed to the potential correlation between the null network and the observed network, coupled with their comparable densities. Specifically, there is a probability of 55.08% of edge overlap between the null network ($l_2$) and the observed network ($l_1$). Furthermore, the observed network contains 305 connections, while the null network contains 440 connections. Adjusting the null network strength to match that of the observed network, according to Eq. (13), produces relatively high $P_{ij}$ values, causing significant changes in the modularity matrix compared to the adjacency matrix of the observed network. Consequently, even minor adjustments to the $\gamma$ value result in a further decomposition of the modules and the formation of numerous small modules.

### Uncover communities with inclusion of overlapping multidimensional edges

In the second case, our objective differs from the previous subsection, as we aim to identify communities characterized by a high degree of overlapping edges. To achieve this, we compare the community structure obtained by maximizing $Q^{inclusion}$ using Eq. (15) with the traditional NG community structure, which is uncovered by maximizing $Q^{conf}$ (Eq. 5).

15 $$\begin{aligned} \varvec{Q}^{inclusion} = \frac{1}{L} \sum \limits _{C \in {\mathcal {C}}} \sum \limits _{ij \in C} \left( A_{ij}^{l_3}-\gamma {\bar{A}}_{ij}^{l_4}\right) \delta \left( c_i,c_j\right) \end{aligned}$$

where the observed network layer is the professional advice network ($l_3$), and the null network layer is the complement graph of the evaluation of the high professional knowledge network ($l_4$) of the same set of nodes. We derive the null network from the directed network that arises from evaluations of high professional knowledge. Our goal is to identify professional communities where expertise is highly valued. In network science terms, this implies communities with a high probability of overlapping edges. The proposed null network reduces the values of the entries in the adjacency matrix of the $l_3$ network layer when computing the modularity matrix, in cases where there is no connection in the $l_4$ network layer.

**Figure 3:**
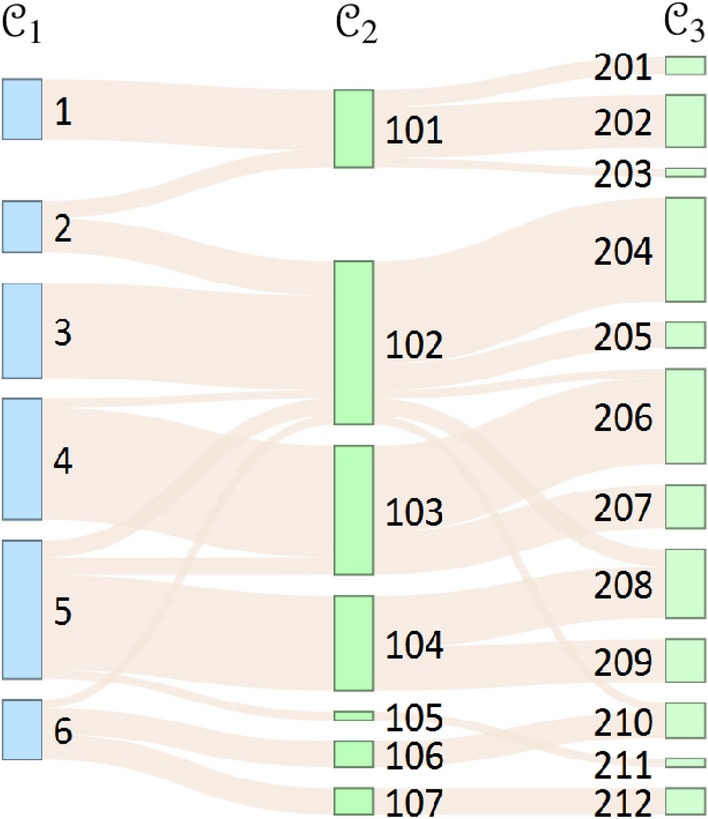
Transformation of communities explored by different null models. The size of the lanes is proportional to the number of people. The null model in community structure ${\mathcal {C}}_1$ (blue) obtained with null model of the configuration model (Eq. 5), the ${\mathcal {C}}_2$ (green) obtained with the adjacency matrix of complement network of $l_4$ layer at $\gamma =1$ (Eq. 15), and the ${\mathcal {C}}_3$ (light green) obtained with stronger consideration at $\gamma =2$ (Eq. 15).

Figure 3 illustrates the transitions in actor placements within modules as we move from the ${\mathcal {C}}_1$ community structure, produced by the NG null model considering Equation 1, to the ${\mathcal {C}}_2$ structure, created by considering Eq. (8) ($\gamma =1$). Additionally, with greater consideration of the null model, it demonstrates how the nodes within the modules of the ${\mathcal {C}}_2$ community structure are further decomposed into the partition ${\mathcal {C}}_3$, considering Eq. (8) with $\gamma =2$. To complement this visual representation, Table 3 provides details on the number of vertices in each module, the local modularity, and the probability of null network edges coinciding with edges in the observed network layer within each module.

**Table 3:** Characterisation of modules shown in Fig. 3. $Q_c$ is the local modularity of the module, and $P(l_4|l_3)$ means the probability of the edges of the null network that occur together with the edges of the observed network within the modules.

${\mathcal {C}}_1$				${\mathcal {C}}_2$				${\mathcal {C}}_3$			
Module id	Number of members	$Q_c$	$P(l_4|l_3)$ within module	Module id	Number of members	$Q_c$	$P(l_4|l_3)$ within module	Module id	Number of members	$Q_c$	$P(l_4|l_3)$ within module
1	6	0.050	0.533	101	9	0.059	0.473	201	2	0.007	0.500
								202	6	0.043	0.571
								203	1	0.000	
2	6	0.045	0.462	102	19	0.203	0.623	204	12	0.099	0.684
3	11	0.087	0.697					205	3	0.021	0.333
4	14	0.097	0.513	103	15	0.122	0.476	206	11	0.074	0.484
								207	5	0.016	0.286
5	16	0.107	0.625	104	11	0.077	0.640	208	6	0.046	0.421
								209	5	0.030	0.889
				105	1	0.000		211	1	0.000	
6	7	0.044	0.692	106	2	0.016	1.000	210	4	0.025	1.000
				107	3	0.019	0.400	212	3	0.017	0.400
		$Q^{{\mathcal {C}}_1}=0.430$				$Q^{{\mathcal {C}}_2}=0.496$				$Q^{{\mathcal {C}}_3}=0.378$	
$Q^{{\mathcal {C}}_1} = Q^{NG}_{l_3} = \frac{1}{L} \sum \limits _{C \in {\mathcal {C}}} \sum \limits _{ij \in C} \left( A_{i,j}^{l_3}-\frac{k^{l_3}_i k^{l_3}_j}{2m}\right) \delta \left( c_i,c_j\right)$				$Q^{{\mathcal {C}}_2} = Q^{inclusion, \gamma =1}_{l_3, l_4} = \frac{1}{L} \sum \limits _{C \in {\mathcal {C}}} \sum \limits _{ij \in C} \left( A_{ij}^{l_3} - 1 {\bar{A}}_{ij}^{l_4}\right) \delta \left( c_i,c_j\right)$				$Q^{{\mathcal {C}}_3} = Q^{inclusion, \gamma =2}_{l_3, l_4} = \frac{1}{L} \sum \limits _{C \in {\mathcal {C}}} \sum \limits _{ij \in C} \left( A_{ij}^{l_3} - 2 {\bar{A}}_{ij}^{l_4}\right) \delta \left( c_i,c_j\right)$			

The combination of Module 1 and the part of Module 2 in the community structure ${\mathcal {C}}_1$ resulted in Module 101 in community structure ${\mathcal {C}}_2$. The degree of edge overlap in Module 101 is lower than in Module 1, but slightly higher than in Module 2. However, the local modularity was increased, considering a different modularity equation. The overlap measure decreases when Module 1 and Module 3 are transforming, while increasing when Module 2 is separating. Furthermore, the local modularity of these modules is increasing. Module 101 is divided into Module 201, Module 202 and Module 203 within the community structure ${\mathcal {C}}_3$, where $\gamma$ increases to 2 in Eq. (15). The overlap of $l_4$ adjacent to $l_3$ in Module 201 and Module 202 is greater than in Module 101. As $\gamma$ increases in Eq. (15), the methodology identifies modules in which the edges within each module have more multidimensional characteristics.

In addition, Table 3 illustrates a strong correlation between local modularity and the size of modules. Smaller modules tend to contribute less to the overall modularity of the community structure, whereas larger modules have a more significant impact.

### Revealing the layers that contribute to the community structure

In this subsection, we demonstrate a different application diverging from the previous two. Initially, we merged the three-layer network and established the NG community structure of the weighted and directed network by maximizing $Q^{NG}$ (Eq. 5) as our baseline reference because there is no ground truth and our objective is not to identify it. We first take note of the shift in the community structure that occurs when $Q^{multi}_{l_1}$ is maximized, which can be expressed formally as follows.

16 $$\begin{aligned} \varvec{Q}^{multi}_{l_1} = \frac{1}{L} \sum \limits _{C \in {\mathcal {C}}} \sum \limits _{ij \in C} \left( A_{ij}^{l_1+l_2+l_3}-\gamma {\bar{A}}_{ij}^{l_1}\right) \delta \left( c_i,c_j\right) \end{aligned}$$

where $A_{ij}^{l_1+l_2+l_3}$ is the adjacency matrix of the observed network which is the merged three-layer network, while the null network was the adjacency matrix of the complement graph of $A^{l_1}$. In this way, the algorithm favored the creation of communities that promote the edges of the $l_1$ layer within the modules. The similarity between the NG and the $l_1$ layer promoted community structure measured by NMI, which is 0.790. The procedure was performed with each layer and each pair of layers preferred. The results are summarized in Table 4.

**Table 4:** The similarity between NG community structure generated by configuration model and community structures obtained by different null network that promote network layer(s) within communities.The $\gamma$ adjusting parameter is 1 in all cases.

Null network promoted network layer	$\varvec{A}$	$\varvec{P}$	Modularity (Q)	NMI
NG $l_1$ + $l_2$ + $l_3$	$A_{ij}^{l_1+l_2+l_3}$	$\frac{k_i k_j}{2m}$	$Q^{NG}$=0.389	1
$l_1$ (Cooperation)	$A_{ij}^{l_1+l_2+l_3}$	${\bar{A}}_{ij}^{l_1}$	$Q^{multi}_{l_1}$=0.461	0.790
$l_2$ (Friendship)	$A_{ij}^{l_1+l_2+l_3}$	${\bar{A}}_{ij}^{l_2}$	$Q^{multi}_{l_2}$=0.461	0.837
$l_3$ (Professional advice)	$A_{ij}^{l_1+l_2+l_3}$	${\bar{A}}_{ij}^{l_3}$	$Q^{multi}_{l_3}$=0.452	0.799
$l_1$ + $l_2$	$A_{ij}^{l_1+l_2+l_3}$	${\bar{A}}_{ij}^{l_1+l_2}$	$Q^{multi}_{l_1+l_2}$=0.461	0.850
$l_1$ + $l_3$	$A_{ij}^{l_1+l_2+l_3}$	${\bar{A}}_{ij}^{l_1+l_3}$	$Q^{multi}_{l_1+l_3}$=0.458	0.829
$l_2$ + $l_3$	$A_{ij}^{l_1+l_2+l_3}$	${\bar{A}}_{ij}^{l_2+l_3}$	$Q^{multi}_{l_2+l_3}$=0.459	0.799

When optimizing $Q^{multi}$ (Eq. 16), there is a preference for the presence of a single layer or multilayer defined by the null network $\varvec{P}$ within the modules, leading to module cuts that ideally minimize the impact on edges in the null network. Despite two distinct optimization approaches (maximizing $Q^{NG}$ and $Q^{multi}$) producing different community structures, their similarity indicates that similar cohesive ”forces” shape the modules, characterized by dense connections. As seen in Table 4, the NMI values suggest that the formation of complex workplace communities, compared to the configuration model, is more influenced by the friendship layer, while the cooperation connections have slightly less influence. The interviews indicated that friendships are an important factor in the functioning of the workplace that was studied.

## Conclusions

We have demonstrated that in the context of multilayer networks, employing one layer as a null network allows us to find communities where edges overlap.

The contribution of this work is a novel modularity measure that reveals meaningful communities based on edge overlap in multiplex networks. We explored the significance of the null network in the context of multilayer networks. As demonstrated in several previous sections, a null network based on a model represents a known information set against which the revealed communities signify a surplus of relationships. Thus, it becomes possible to selectively ”remove” the desired information from the observed network with the null network. Our work reinforces this concept through the application of an empirical null network.

There exists an intriguing duality when investigating modules. The modularity matrix uses the null network to weaken the *edges* in the observed network, allowing play with the strength of the edges while determining the membership of *nodes* in the modules. During the clustering process, the null network identifies weak ties within the observed network. It offers a viewpoint in which certain connections appear weakened, while others retain their strength, influenced by the null network. When maximizing modularity, the links weakened by the null network may become cutting points that separate nodes to modules. Meanwhile, strong links persist within the modules, contributing significantly to the overall modularity value (Q). In Eq. (7), the modularity matrix classifies the links of the observed network that overlap with the links of the null network as weak. In contrast, the modularity matrices defined in Eqs. (8) and (11) classify the overlapping links as strong. Essentially, Eq. (7) increases, whereas Eqs. (8) and (11) decrease the likelihood of break points along the overlapping edges.

In the first empirical case (illustrated by Eq. (7)), we employed a network layer as a null network to eliminate the information set from the communities coded by the network layer, resulting in relatively low layer overlaps within them. In the second empirical case, we subtract the information set of a complement graph of network layer as null network from the observed network, effectively retaining the connections of the observed network layer that overlap with the null network, which allows us to obtain communities with high likelihood of edge overlaps within them. In the third case of merged multilayer networks, a community structure can be displayed where the appearances of individual layers within communities are favored.

As a minor recognition, our empirical evidence indicates that the edge density of the null network influences the degree of interference in community formation by the benchmark network. (1) When searching for modules based on a null network layer. The edge density of the null network closely approximates that of the observed network. Consequently, when we align the strengths according to Eq. (13), we obtain high values for $P_{ij}$, while the modularity matrix displays low values where subtraction occurs. This leads to a significant alteration in the community structure, particularly with an increase in the value of $\gamma$. However, the algorithm aims to exclude edge overlaps within communities, which can also result in significant changes, since edge overlaps may be correlated. (2) When we utilized the complement network as the null network, characterized by high density, the values of $P_{ij}$ are significantly lower when the strengths are set equal to those of the observed network. Consequently, the values of the modularity matrix closely resemble those of the adjacency matrix of the observed network, reducing the disruptive influence of the null network. An increase in the $\gamma$ value results in only minor changes, as shown in Fig. 3.

We have demonstrated the practical significance of this approach using a network of organizational coworkers interconnected in various aspects. However, this method can be applied to study any system that can be modeled as a multilayer network, particularly in cases where it is advantageous to identify cooperative communities that collaborate in multiple aspects or not.

### Supplementary Information

Supplementary Information.

## Data Availability

The raw data analyzed during this study are anonimized and available on Github^34^.
