# Supplementary material for: Finding multifaceted communities in multiplex networks
Source: Sci Rep. 2024 Jun 24;14:14521. doi: 10.1038/s41598-024-65049-6 (PMC11196740; doi:10.1038/s41598-024-65049-6)
Supplement: Supplementary file 1 — Supplementary Information. [file 41598_2024_65049_MOESM1_ESM.pdf]

Supplementary materials

The method was tested on an additional small (61 nodes, 194 edges) and one large (1909 nodes, 28,586 edges) database. Feasible research questions were used to explore object-oriented communities. The aim was to uncover somewhat meaningful communities. The research questions are intended only to demonstrate the scalability, reproducibility of the method, and show the new modularity measure how works with additional datasets.

Tests with AUCS Dataset

The AUCS dataset[53] is a multilayer network of an academic community at Aarhus University where edges represent multifaceted relationships between members. The data set contains undirected and unweighted relationships of 61 employees and leaders of four different connection types. Visualizations of the network highlighting the modules and analysis codes are available on the GitHub website[34] linked to this article.

Community detection with exclusion of overlapping edges

The aim of the modularity measure used is to explore groups of collaborators who work together but have not yet published together. These groups reflect communities of colleagues for whom the working relationship could develop into coauthorship. The module search avoids overlapping coauthorship relationships with working relationships. That is, $\boldsymbol{A} = A_{work}$, and $\boldsymbol{P} = A_{coauthor}$ in Equation 7.

In a network without administrative staff and actor U71, who is also probably an administrator, there is a 16.82% probability that coauthorship relationships appear alongside work relationships. Our algorithm aims to reduce the probability of this overlap measure within communities. Module detection was carried out with the Louvain algorithm using Equation 7 with $\gamma = 1$.

A large community and several smaller modules were obtained, and multiple single-node modules were revealed. The results are given in Table 5, which does not contain one-node modules. Overall, it can be concluded that the desired goal was achieved and that very few overlapping coauthorship relationships are found within the modules with working relationships. The communities are visualized in Figure 4.

	N	E_{work}	P(coauthor ∣ work)
network	54	107	0.1682
in modules		74	0.0135
between modules		33	0.5152
Module 2	27	62	0.0161
Module 3	3	2	0
Module 5	2	1	0
Module 6	5	5	0
Module 7	2	1	0
Module 14	3	2	0
Module 15	2	1	0

Table 5. The result of edge overlap exclusion method on AUCS data set. N: number of nodes, E_{work}: number of work relationships, P(coauthor ∣ work): the probability that the coauthor relationships overlapped with the work connections

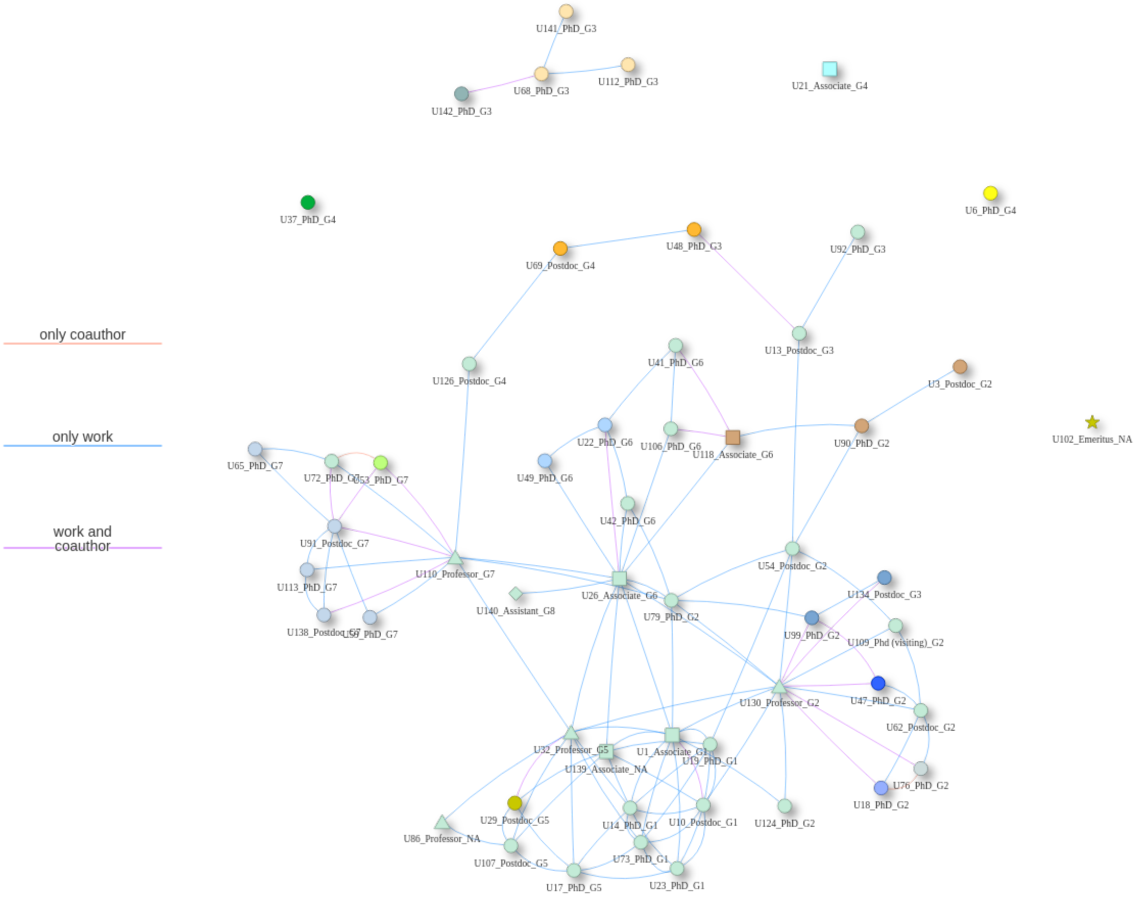

Figure 4. Multilayer network of actors in AUCS dataset where edges represent working and/or coauthor relationships. Nodes are colored by communities. The interactive figure is available online[34].

Community detection with inclusion of overlapping edges

We test the overlapping edge including algorithm by exploring communities in which lunch connections appear alongside work connections. The modules explore meaningful groups of collaborators who work together and also have lunch together. This analysis aims to identify lunch groups in which members of the groups have working relationships.

The purpose of using the complement graph contributing to modularity measure is to ensure that the modularity maximization algorithm prefers those links that overlap within modules. Thus, single edges are preferred for cutting points by the algorithm. In equation 8, $\boldsymbol{A} = A_{work}$ and $\boldsymbol{P} = \bar{A}_{lunch}$, the complement network of the relationship layer A_{lunch} and $\gamma = 2$.

The results are given in Table 6, which does not contain modules with one node. The edge overlap inclusion method reveal modules with high edge overlap within modules. The communities are visualized in Figure 5.

	N	E_{work}	P(lunch | work)
network	61	194	0.5052
in modules		107	0.7477
between modules		87	0.2069
Module 2	11	33	0.7576
Module 3	10	25	0.7600
Module 4	7	22	1.0000
Module 5	8	28	0.7857
Module 8	10	36	0.5556
Module 9	2	1	1.0000
Module 10	2	1	1.0000
Module 11	6	4	0.5000

Table 6. The result of edge overlap inclusion method on AUCS data set. N: number of nodes, E_{work}: number of work relationships, P(lunch | work): the probability that lunch relationships overlapped with work connections

Tests with Erasmus student network

The size of the Erasmus network (1909 nodes, 28,586 edges) is large enough to infer the scalability of the method. In the Erasmus student network, the nodes represent higher education institutions (HEIs), and the weighted and directed edges depict long-term student travels between them. These edges possess a disciplinary dimension, making the network a multilayer structure[54]. Although the majority of edges are one-dimensional, related to one subject, HEIs often span multiple disciplines[36]. For the purpose of analysis, the links were simplified to an undirected and unweighted form.

To demonstrate the methodology, the HEIs were grouped according to the dimensionality of the links. Specifically, we selected science, technology, engineering and mathematics (STEM) professions as the student travel dimension and investigated the groups according to the diversity of connections. Using the exclusion method, we explored groups where engineering-type trips exhibited higher density within modules compared to between modules, while overlapped IT-type links were rare. In contrast, with the inclusion method, we examined the opposite scenario and identified groups where IT-type connections overlapped next to engineering-type connections with high probability.

The NMI value comparing the community structures obtained from the exclusion and inclusion methods is relatively high, at 0.575. This is mainly because both methods produced a large community. More than half of the nodes (187 out of 285) that belong to Module 5 resulted from the inclusion method are also present in Module 4 obtained by the exclusion method. This indicates a densely connected region of the network that is not partitioned by either method. However, the affiliated HEI of the authors, University of Pannonia, exhibits a different pattern. Completely different institutions are found in its modules obtained by the exclusion and inclusion methods.

Community detection with exclusion of overlapping edges

The identified groups represent communities of HEIs characterized by engineering relationships but lacking overlapped science, mathematics, and computing (IT) relationships. To achieve this, $\boldsymbol{A} = A_{engineering}$, and $\boldsymbol{P} = A_{IT}$ in Equation 7 were used.

Within the network, there is a probability of 21. 22% that IT relationships appear alongside engineering relationships. This method aims to reduce the probability of overlap within communities. Module detection was conducted using the Louvain algorithm with Equation 7 where $\gamma = 2$.

A very large community and several smaller communities were obtained. The results, presented in Table 7, include modules consisting of 20 or more nodes, although a total of 445 communities were identified. Few IT relationships are found to overlap within the modules with engineering relationships.

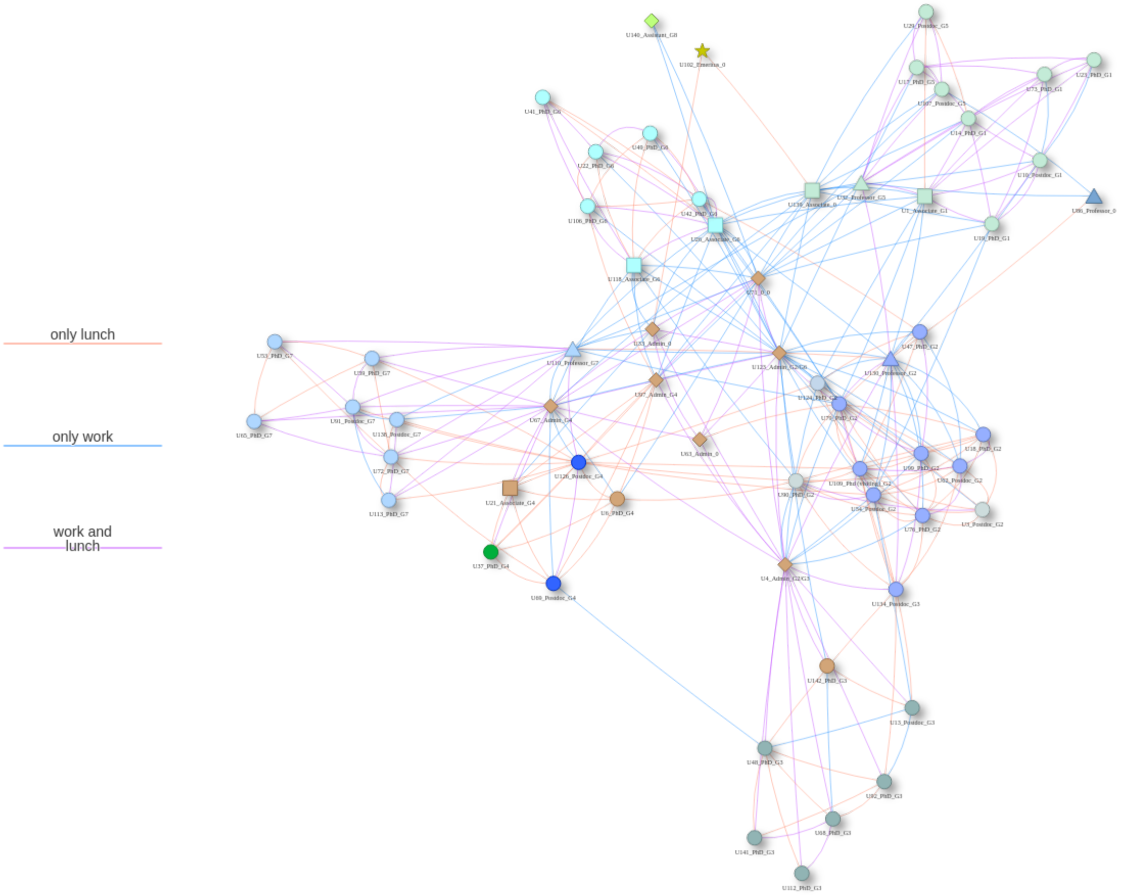

Figure 5. Multilayer network of actors in AUCS dataset where edges represent working and/or lunch relationships. Nodes are colored by communities. The interactive figure is available online[34].

	N	E_{engin}	P(IT|engin)
network	1,909	28,586	0.2122
in modules		14,512	0.1192
between modules		14,074	0.3080
Module 3	46	44	0.000
Module 4	933	13,676	0.1258
Module 6	127	168	0.0417
Module 7	48	42	0.0000
Module 8	62	68	0.0294
Module 9	342	43	0.0000
Module 10	72	65	0.0000
Module 14	20	23	0.0000
Module 17	24	18	0.0000
Module 19	35	39	0.0000
Module 22	26	34	0.0000
Module 30	30	1	0.0000
Module 42	27	32	0.0000

Table 7. The result of edge overlap exclusion method on Erasmus student network. N: number of nodes, E_{egin}: number of engineering relationships, P(IT|engin): the probability that the IT relationships overlapped with the engineering connections

Community detection with inclusion of overlapping edges

The modules explore meaningful groups of higher education institutions (HEIs) that cooperate in the field of engineering and also have overlapping connections in the field of IT, reflecting STEM multidisciplinarity.

In equation 8, $\boldsymbol{A} = A_{engineering}$ and $\boldsymbol{P} = \bar{A}_{IT}$, the complement network of the relationship layer A_{IT} and $\gamma = 10$ was used in this method. The reason for the high γ value is that the IT network is sparse (0. 81%), resulting in a very dense complement network $\boldsymbol{P}$. When we bring the adjacency matrices $\boldsymbol{A}$ and $\boldsymbol{P}$ to the same strength (see Equation 13), the values of $\boldsymbol{P}$ will approach close to 0, which explains some disfunction.

The results are given in Table 8, which contains modules with 10 or more nodes. In total, there are 810 modules. The method provides a large number of small communities. However, considering that a large proportion of the relationships are one-dimensional, it may be reasonable to conclude that there are no large groups with a high probability of edge overlaps.

	N	E_{engin}	P(IT ∣ engin)
network	1,909	28,586	0.2122
in modules		13,846	0.2623
between modules		14,740	0.1650
Module 3	14	43	0.1163
Module 5	285	12,366	0.2722
Module 17	21	75	0.1067
Module 22	15	54	0.2037
Module 23	10	14	0.0714
Module 24	12	25	0.0000
Module 54	11	15	0.4667
Module 56	13	36	0.0833
Module 69	14	48	0.04167
Module 73	10	12	0.5833
Module 76	13	25	0.1600
Module 77	13	33	0.1515
Module 153	10	9	0.0000
Module 316	11	10	0.2000

Table 8. The result of edge overlap inclusion method on Erasmus student network. N: number of nodes, E_{egin}: number of engineering relationships, P(IT ∣ engin): the probability that IT relationships overlapped with engineering connections
